# Magnetic Resonance Imaging-guided Brachytherapy Re-irradiation for Isolated Local Recurrence of Soft Tissue Sarcoma

**DOI:** 10.7759/cureus.2457

**Published:** 2018-04-10

**Authors:** Noelia Sanmamed, Alejandro Berlin, Akbar Beiki-Ardakani, Heather Ballantyne, Anna Simeonov, Peter Chung

**Affiliations:** 1 Radiation Oncology, Princess Margaret Cancer Centre, Toronto / University of Toronto, Toronto, CAN

**Keywords:** sarcoma, recurrence, mri-guided, brachytherapy

## Abstract

Management options for locally recurrent sarcoma of the pelvis in patients previously treated with external beam radiation and surgery are limited. Pelvic exenteration is often the only viable but unattractive option. We present a patient with recurrent myxoid round cell liposarcoma of the ischiorectal fossa treated in 2013 with preoperative radiation (50 Gy in 25 fractions) and subsequent wide local resection. Four years later, a follow-up magnetic resonance imaging (MRI) scan demonstrated a 1 x 1 cm T2 hypointense soft tissue pre-sacral nodule consistent with local recurrence (LR). The patient declined posterior pelvic exenteration and was treated with 12 Gy using high dose rate brachytherapy (BT) under MRI-guidance followed by a further external beam radiation to a dose of 30 Gy in 15 fractions.

## Introduction

Soft tissue sarcoma (STS) represents <1% of all adult malignancies [1]. Surgery and radiation are the most common primary approaches for patients with localized disease. Despite radical treatment, local recurrence (LR) in STS ranges from 5% to 20%. Myxoid liposarcoma (MLS) is relatively radiosensitive compared to other STS, with five-year local control rates reported to be around 97% [2]. LR in patients who have received previous radiation continues to be a challenge, as re-treatment with a combined approach is often not feasible. Secondary to the increased morbidity of re-irradiation with external beam radiotherapy (EBRT) in this setting, the main therapeutic option is radical surgery alone, which may involve limb amputation or pelvic exenteration. Brachytherapy (BT) offers the possibility of delivering high doses in the target volume with rapid dose fall-off in the surrounding normal tissue that may minimize radiation toxicity [3]. For STS, magnetic resonance imaging (MRI) provides high-resolution imaging for optimal soft tissue definition compared with ultrasound (US) or computed tomography (CT), especially in a patient with altered anatomy after previous surgery and radiation.

## Case presentation

**Figure 1:**
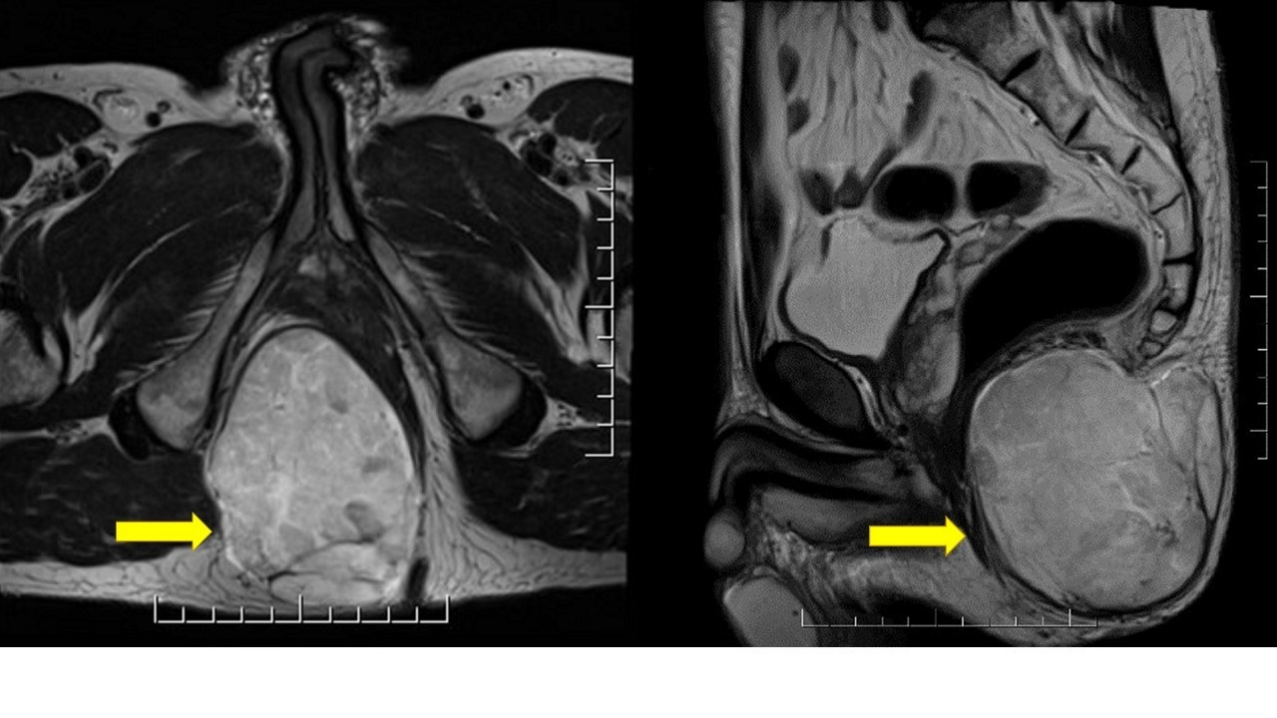
Axial and sagittal T2-weighted magnetic resonance imaging (MRI) sequences showing a multilobulated mass with heterogeneous enhancement

A 43-year-old man presented with a lesion in the ischiorectal fossa. An MRI scan demonstrated an enhancing, solid mass measuring 6.2 x 9.0 x 9.5 cm, right posterior to the anus. (Figure 1).

The biopsy was positive for grade 2 MLS with 5% round cell component. Staging investigations including CT of the thorax, abdomen, and pelvis were negative for metastatic disease.

In order to avoid an abdomino-perineal resection (APR), neoadjuvant chemotherapy and radiation were offered to the patient. He refused chemotherapy and he was treated with radiotherapy alone, 50 Gy in 25 fractions. Repeat CT and MRI studies six weeks after neoadjuvant radiotherapy demonstrated a reduction in the size of the mass from 9.8 x 9.6 x 6.7 cm to 6.4 x 6.2 x 2.9 cm.

However, APR remained the best option for definitive surgery, which the patient declined. He subsequently underwent a wide local excision with partial resection and reconstruction of the anal sphincter. 

**Figure 2:**
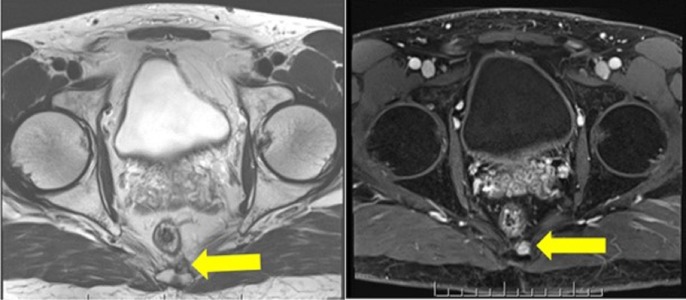
Axial T2-weighted (left) and DCE MRI (right) sequences showing a nodule located left and anterior to the lower coccygeal segment, which demonstrates intermediate hypointensity in T2 and DCE abnormal enhancement DCE: dynamic contrast-enhanced; MRI: magnetic resonance imaging.

He remained disease-free for four years until surveillance MRI found a new soft tissue nodule (1 x 1 cm) anterior to the coccyx, suspicious for local recurrence (Figure 2). A biospy was recommended but declined by the patient.

No evidence of metastatic disease was found on staging work-up. Surgical resection (APR with coccyx resection) was recommended, but this was declined by the patient. In spite of the fact that the recurrence was located in the previously radiated volume, re-irradiation with high-dose rate brachytherapy (HDR-BT) followed by limited EBRT was considered and the patient accepted this treatment plan. 

Prior to the planned BT procedure, a pre-planning/simulation study using transrectal ultrasound (TRUS) was attempted. However, secondary to the altered anatomy, post-treatment fibrosis and significant discomfort of the patient, the US-probe could not be inserted. As a result, the BT procedure was done in an MR-guided interventional suite, taking into account the potential need for MR-directed brachytherapy. Our dedicated interventional MRI suite consists a 1.5 Tesla MRI scanner on rails that moves between a linear accelerator (Varian, True beam) and the HDR brachytherapy suite. It was custom designed to allow MR-guidance for advanced radiotherapy management. The suite includes a “radio frequency isolation equipment room” to allow MR-incompatible equipment (e.g., HDR afterloader, fluoroscopy unit and ultrasound) to remain energized during MR-based procedures [4].

**Figure 3:**
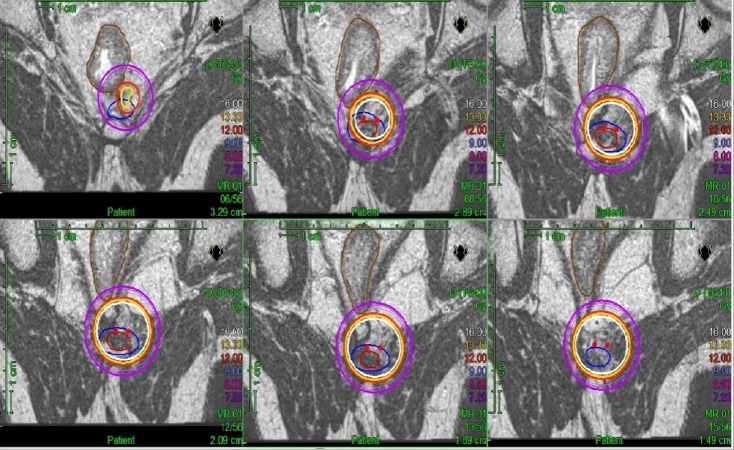
T2-weighted planning MR images (FOV 180 mm, TR 4400 ms, TE 103 ms, 2 mm slice thickness) demonstrating brachytherapy dose distribution in Gy GTV in red, PTV in blue, rectum in brown. MR: magnetic resonance; FOV: field-of-view; TR: repetition time; TE: echo time; GTV: gross tumor volume; PTV: planning tumor volume.

The patient was positioned on an interventional MR tabletop (InVivo Inc) in modified lithotomy position. General anesthesia was induced and a TRUS probe was inserted into the rectum to guide brachytherapy catheter insertion. However, due to the small size of the lesion and the proximity to the coccyx, visualization and accurate localization of the tumour was not possible. Therefore, we converted to catheter insertion under MRI guidance. A sterile custom MR-compatible perineal template was fixed to the tabletop and proton density weighted images were obtained to guide catheter positioning. Two brachytherapy catheters were inserted into the target volume. After the implant was complete, final high-resolution two-dimensional T2-weighted images were obtained and the gross tumor volume (GTV) was delineated. Rectum was contoured as an organ-at-risk (OAR) and the planning tumor volume (PTV) was formed by adding 5 mm margin beyond the GTV restricted to the OAR. A BT plan was generated using Oncentra planning system (Elekta). Target coverage and dose volume histogram were evaluated and optimized to achieve adequate dosimetry. PTV dose was 12 Gy in a single fraction (PTV D95 12.2 Gy, GTV D99 16.3 Gy) was delivered with remote after loading (Figure 3).

For EBRT, a volumetric modulated arc therapy plan was generated after CT-MRI fusion for treatment planning. We created the clinical target volume (CTV) by expanding the GTV with a 5 mm margin circumferentially and up to 2 cm inferiorly, excluding the rectum. The PTV was formed adding 5 mm to the CTV. A total of 30 Gy in 15 fractions was delivered and image-guidance was with cone-beam CT imaging. The dose was chosen empirically based on the previous external beam dose of 50 Gy in 25 fractions that had already been delivered. It was felt a dose total dose of 80 Gy external beam radiation would be associated with potentially acceptable risk of long-term toxicity in addition to the HDR brachytherapy dose.

The patient tolerated the treatment well but described mild perineal discomfort (Common Terminology Criteria for Adverse Events (CTCAE) v4.0 Grade 1) after the brachytherapy procedure which did not require analgesia that resolved spontaneously within a week. Follow up thus far is limited but further follow up clinically and with MRI will be performed for toxicity and response assessment.

## Discussion

Sarcoma is an uncommon and heterogenous group of malignancies. The benefit of radiotherapy in the treatment of STS is well-established. Our patient declined to undergo further non-organ preserving surgery, thus an alternative management plan was required. HDR-BT delivers higher dose to the tumor while minimizing dose to the adjacent normal tissue, reducing toxicity risk without compromising the dose to the target volume. BT has been used by some groups in sarcoma re-irradiation with five-year local control and overall survival of more than 50% [5-6]. Limited dose EBRT may be added to supplement inherent dose heterogeneity associated with brachytherapy and to extend coverage in areas suspected of bearing microscopic disease. US-based guidance for treatment in our patient presented challenges due to the proximity of the rectum and inability of US to produce adequate images for catheter insertion. Alternatively a CT-based approach to the brachytherapy procedure might be considered more consistent with 'standard-of-care'; however, our institution currently does not have the ability for patients to undergo CT-based procedures under general anaesthesia. As we had the availability of an interventional MR suite, the MR-guided approach was ideal in this situation as the tumour was easily visualized on non-contrast MR sequences, allowing needle insertion directed to the target area. The patient could be treated without having to be transferred to a separate brachytherapy suite. While both CT and MR would be expected to be similar for target and OAR delineation, our view is that the superiority of soft tissue contrast in MRI confers an advantage for both target and OAR delineation in this case, even though this may not necessarily result in a measurable clinically meaningful difference. Brachytherapy dosimetry would be expected to be similar regardless of the imaging modality used for treatment planning with our brachytherapy planning system.

## Conclusions

MRI allowed accurate localization and delineation of locally recurrent STS. Moreover, it enabled the use of brachytherapy for salvage treatment. Longer follow-up is needed to evaluate late toxicity and potential benefit of treatment in this particularly challenging case.
